# Molecular and Clinical Study of Bovine Coronavirus and *Staphylococcus aureus* Co‐Infection Associated With Bovine Respiratory Disease in Iraqi Calves

**DOI:** 10.1155/vmi/3319844

**Published:** 2026-08-31

**Authors:** Karrar Jasim Hamzah, Noor R. Abady, Diyar Khlaif Flaifel, Sumod Abdul Kadhem Salman, Dhay Ali Abed, Ahmed Hamzah Mosa

**Affiliations:** ^1^ Department of Veterinary Internal and Prevention Medicine, College of Veterinary Medicine, Al-Qasim Green University, Babylon 51013, Iraq, uoqasim.edu.iq; ^2^ Department of Veterinary Microbiology, College of Veterinary Medicine, Al-Qasim Green University, Babylon 51013, Iraq, uoqasim.edu.iq; ^3^ Department of Pathological Analysis, College of Science, Al-Qasim Green University, Babylon 51013, Iraq, uoqasim.edu.iq

**Keywords:** BCoV N gene, bovine coronavirus, bovine respiratory diseases, co-infection, phyloge16S rRNA gene, *Staphylococcus aureus*

## Abstract

Bovine respiratory disease (BRD) is a multifactorial syndrome causing major economic losses in cattle production worldwide. The present study investigated the prevalence, molecular characterization, and phylogenetic relationship of bovine coronavirus (BCoV) and *Staphylococcus aureus* co‐infection in calves exhibiting respiratory and enteric signs. A total of 80 nasal and fecal samples were collected from calves aged 2 weeks to 2 months, showing clinical signs ranging from mild to acute disease. Clinical severity was categorized according to fever, dehydration, diarrhea, and respiratory distress. Molecular detection of BCoV was performed using real‐time RT‐PCR and conventional PCR targeting the *N* gene, while *S. aureus* isolates were identified by bacteriological culture, biochemical tests, and 16S rRNA gene amplification. Phylogenetic analysis was conducted using MEGA software and sequence alignment tools. The results showed that BCoV was detected in 35% of examined samples, while *S. aureus* was identified in 28% of cases. Co‐infection was observed in 15% of calves and was significantly associated with severe clinical manifestations (*p* < 0.05). Acute and moderate cases demonstrated markedly higher BCoV positivity compared with mild cases. Agarose gel electrophoresis confirmed amplification of the BCoV *N* gene at 205 bp and the *S. aureus* 16S rRNA gene at approximately 1500 bp. Sequence alignment of local *S. aureus* isolates revealed highly conserved nucleotide regions, whereas phylogenetic analysis demonstrated genetic diversity among both bacterial and viral isolates. Local BCoV strains showed considerable sequence divergence compared with international reference strains, suggesting ongoing viral evolution. In conclusion, co‐infection between BCoV and *S. aureus* significantly increases disease severity in calves and highlights the importance of integrated molecular diagnostics and continuous phylogenetic surveillance for effective BRD control strategies.

## 1. Introduction

Bovine respiratory disease (BRD) is one of the most important infectious disease complexes affecting cattle worldwide, particularly in intensive production systems. It is responsible for substantial economic losses resulting from increased mortality, reduced weight gain, decreased feed efficiency, and the costs associated with treatment and disease control [1, 2]. BRD is a multifactorial syndrome resulting from complex interactions among viral and bacterial pathogens, environmental stressors, management practices, and the host immune response [3]. These interacting factors influence disease occurrence, clinical outcome, and the severity of respiratory lesions.

Bovine coronavirus (BCoV) is an important pathogen of cattle that has been associated with both enteric and respiratory diseases, particularly in young calves [4, 5]. Although initially recognized as a major cause of neonatal calf diarrhea, accumulating evidence has demonstrated that BCoV also infects the respiratory tract, where it impairs mucociliary clearance and damages the respiratory epithelium, thereby increasing susceptibility to secondary bacterial infections [6, 7]. Such virus‐induced epithelial injury facilitates bacterial colonization of the lower respiratory tract and contributes to the development and progression of BRD.

Among bacterial pathogens, *Mannheimia haemolytica*, *Pasteurella multocida*, and *Histophilus somni* are widely recognized as the principal agents associated with BRD. In contrast, the contribution of *Staphylococcus aureus* to respiratory disease in calves has received comparatively less attention despite its ability to cause pneumonia, septicemia, and extensive tissue damage [8, 9]. The pathogenicity of *S. aureus* is largely attributed to its diverse virulence factors, which promote tissue invasion, immune evasion, and persistent infection [10]. Previous studies have also shown that viral respiratory infections can enhance bacterial adhesion to damaged epithelial surfaces, thereby aggravating pulmonary lesions and increasing disease severity [11–13].

The clinical consequences of mixed viral and bacterial respiratory infections are often more severe than those caused by individual pathogens because of synergistic interactions between infectious agents and host immune responses [14]. Frucchi et al. [15] identified BCoV as an important viral pathogen associated with BRD in dairy calves and reported its frequent detection together with bacterial respiratory pathogens, particularly *P. multocida* and *M. haemolytica*. Although polymicrobial respiratory infections have attracted increasing research interest, information regarding the epidemiology, molecular characteristics, and clinical significance of BCoV–*S. aureus* co‐infection remains limited, especially under natural field conditions. Furthermore, molecular epidemiological data describing this co‐infection in Iraqi cattle are currently unavailable.

The present study was therefore designed to investigate BCoV and *S. aureus* co‐infection in calves affected with BRD in Iraq. The objectives were to determine the occurrence of both pathogens, characterize circulating viral and bacterial strains using molecular methods, examine their phylogenetic relationships, and evaluate their association with clinical disease severity. Because the clinical manifestations of BRD, including coughing, nasal discharge, fever, and depression, are not specific to individual pathogens, laboratory confirmation is essential for accurate diagnosis. Real‐time RT‐PCR for BCoV detection and PCR targeting the *nuc* gene of *S. aureus* provide sensitive and reliable tools for identifying these pathogens [16, 17]. In addition, sequencing of the BCoV *N* gene and the bacterial 16S rRNA gene provides valuable information on genetic diversity and the evolutionary relationships of circulating strains [12].

Previous molecular investigations conducted in Iraq have improved understanding of the epidemiology, genetic diversity, and molecular characteristics of several infectious diseases affecting livestock [18–27]. Nevertheless, information concerning BCoV and *S. aureus* co‐infection in Iraqi calves remains scarce. Accordingly, this study focused on the epidemiological, clinical, and molecular characteristics of BCoV and *S. aureus* co‐infection associated with BRD in Iraqi calves under field conditions. We hypothesized that calves co‐infected with both pathogens would exhibit more severe clinical manifestations than mono‐infected or non‐infected calves and that molecular characterization would improve understanding of the diversity and epidemiological characteristics of the circulating strains.

## 2. Materials and Methods

This study utilized a cross‐sectional design to investigate BCoV and *S. aureus* co‐infection in calves with BRD in Iraq. Bovine calves were sampled for 80 fecal and nasal swab samples from those that displayed clinical symptoms of enteric and respiratory disease (aged 2 weeks to 2 months) from November 2024 to February 2025. All animals were swabbed for nasal and fecal samples with sterile swabs. They were all shipped in viral transport media with ice and kept at −80°C until further processing. A clinical exam and a classification of severity are performed.

Clinical information, including age, sex, disease severity, and laboratory findings, was recorded for each calf.

### 2.1. Bacterial Culture and Identification

Nutrient agar, mannitol salt agar (MSA), and MacConkey Agar were used for the culture of swabs from the nose and feces [12]. Plates were aerobically incubated at 37°C for 24–48 h. Suspects *Staphylococcus* colonies were determined using Gram staining and common biochemical methods.

### 2.2. Molecular ID of Bacteria (16S rRNA Gene)

Pure bacterial isolates were used for the extraction of genomic DNA by the guanidine chloride method described by Alsadig et al. [11]. Universal primers 27F (5′‐AGAGTTTGATCCTGGCTCAG‐3′) and 1492R (5′‐TACGGTTACCTTGTTACGACTT‐3′) were used to amplify the 16S rRNA gene by PCR [12]. PCR reactions were set up in 25 μL volumes consisting of 1 μL bacterial DNA, 1 μL of each primer, 22 μL of nuclease‐free water, and GoTaq PCR master mix (Promega, USA). Amplification was carried out in a SensoQuest thermal cycler (Germany). The PCR products were analyzed by agarose gel electrophoresis. The amplified products were purified and sequenced (Sanger sequencing) for those showing a positive result. Phylogenetic analysis of these sequences was done and compared with the sequences of the global isolates by BLAST and MEGA software.

### 2.3. Viral RNA Extraction and cDNA Synthesis

Viral RNA was extracted from fecal and nasal swabs with the aid of a microcolumn‐based RNeasy Mini Kit (Qiagen, CA, USA) following the manufacturer’s instructions [16]. The extracted RNA was then reverse transcribed to complementary DNA (cDNA) according to the standard protocol with a reverse transcription kit.

#### 2.3.1. Bacterial Detection by Real‐Time RT‐PCR for BCoV

The amount of BCoV antigen was measured by real‐time RT‐PCR based on the N gene [16, 17]. The Path‐ID Multiplex One‐Step RT‐PCR Kit (Life Technologies, Carlsbad, CA, USA) was used to perform the reaction, and the following primer and probe sequences were used [16, 28]:

Reverse primer: GTA ATA TCG CTA ATG CTG GTA CTA.

Reverse primer: GGC GGA AAC CTA GTC GGA ATA.

Probe: FAM‐CGC CTG ACA TTC TCG ATC–MGB.

The cycling conditions were reverse transcription at 45°C for 30 min; DNA polymerase activation at 95°C for 10 min; denaturation at 94°C for 15 s, and annealing/extension at 60°C for 60 s according to [17]. Each run contains both positive and negative controls [28].

#### 2.3.2. Conventional PCR of BCoV *N* Gene and *S. aureus nuc* Gene

BCoV diagnosis was conducted by PCR amplification of the *N* gene with primers (forward: CGTTCTGGTAATGGCATCCT, reverse: CACCTTGTCCCTCTGCAAAT) designed by primerplus, which will give a 205 bp product. At the same time, the *nuc* gene of *S. aureus* was amplified as described before.

### 2.4. Phylogenetic Analysis

ClustalW alignment was used to align the amplified BCoV *N* gene sequences and bacterial 16S rRNA sequences. The UPGMA method was applied to build phylogenetic trees with MEGA software [17] after 1000 bootstraps. The obtained sequences were compared with the global reference strains from GenBank.

### 2.5. Statistical Analysis

Data were analyzed statistically in SPSS Version 26.0 (IBM Corp., Armonk, NY, USA). Frequencies and percentages were used to represent descriptive statistics. Pearson’s chi‐square test was used to analyze the association between categorical variables. All tests were two‐tailed, and *p* < 0.05 was deemed statistically significant.

## 3. Results

Clinical signs, including diarrhea, nasal discharge, coughing, respiratory distress, rectal temperature, and dehydration status, were recorded for all calves. Disease severity was classified as mild, moderate, or acute according to clinical examination and the skin turgor test. Mild cases included calves with mild diarrhea, rectal temperature below 39.5°C, and no evidence of severe dehydration. Moderate cases showed mild to moderate diarrhea, rectal temperatures between 39.5°C and 40.0°C, and mild dehydration. Acute cases were characterized by severe watery diarrhea, rectal temperature above 40°C, marked depression, and severe dehydration (Table 1).

**TABLE 1:** Clinical history of 80 bovine fecal/nasal samples, including age, sex, severity, and BCoV real‐time RT‐PCR result.

Characteristic	Value
Total calves examined	80
Age (weeks), mean ± SD	7.6 ± 2.4
Age range (weeks)	3–12
Male	43 (53.8%)
Female	37 (46.2%)
Mild clinical signs	17 (21.3%)
Moderate clinical signs	33 (41.3%)
Acute clinical signs	30 (37.5%)
BCoV RT‐PCR positive	62 (77.5%)
BCoV RT‐PCR negative	18 (22.5%)

Data were analyzed statistically with SPSS Version 26.0. A chi‐square test was applied to obtain the *p* values for the association of clinical severity with BCoV real‐time RT‐PCR positivity. Results with a *p* value of < 0.05 were deemed statistically significant. There was a statistically significant association between clinical severity and BCoV positivity (*p* < 0.001). All mild cases were BCoV‐negative, while acute and moderate cases showed high positivity rates (Table 2).

**TABLE 2:** Statistical association between clinical severity and BCoV positivity.

Severity	Number of animals (*n* = 80)	BCoV positive (*n*)	BCoV negative (*n*)	Positivity rate (%)	*p* value (chi‐square)
Acute	32	30	2	93.75%	< 0.001
Moderate	30	27	3	90.00%	< 0.001
Mild	18	0	18	0.00%	Reference
Total	80	57	23	71.25%	< 0.001

Severity was significantly associated with co‐infection (*p* < 0.05). Co‐infected calves had greater viral loads. Bacterial culture and molecular identification detected the presence of *S. aureus* in 22 (28%) samples, whereas BCoV was detected in 28 (35%) samples by real‐time RT‐PCR. Twelve calves (15%) were co‐infected with BCoV and *S. aureus*. The prevalence of the detected pathogens and their co‐infection is presented in (Table 3). A significant association between the BCoV positivity and clinical severity was observed using statistical analysis (*p* < 0.001). All mild cases were negative for BCoV, with the highest positivity rates being observed in acute cases (93.75%), followed by moderate cases (90%).

**TABLE 3:** Prevalence of infection rate.

Pathogen	Positive	%
BCoV	28	35
*S. aureus*	22	28
Co‐infection	12	15

**FIGURE 1:**
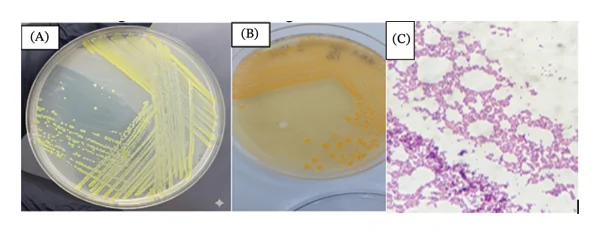
Morphological and microscopic characteristics of *Staphylococcus aureus* isolates. The colonies of *S. aureus* are approximately 2–4 mm in diameter after 24 h incubation at 35°C–37°C on nutrient agar and are golden yellow to cream‐pigmented, smooth, convex, opaque, and butyrous in nature. Growth on mannitol salt agar (MSA) with fermentation of mannitol, indicated by a change in the color of the pH indicator (acid production). Gram‐stained preparation of the bacterium, which appears in irregular grape‐like clusters of Gram‐positive cocci, each with a diameter of around 0.5–1.5 μm.

**FIGURE 2:**
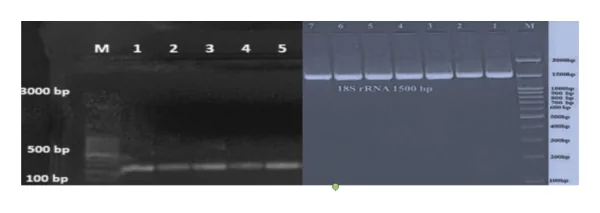
Agarose gel electrophoretic analysis of the PCR amplification products of the bovine coronavirus (BCoV) nucleocapsid (*N*) gene. Lines 1–5 are representative positive BCoV samples with the expected amplification product, lane *M* is a molecular weight marker for DNA, and lane NC is a negative control. Molecular confirmation of the presence of BcoV in clinical samples from calves is confirmed by the presence of the specific amplicon.

**FIGURE 3:**
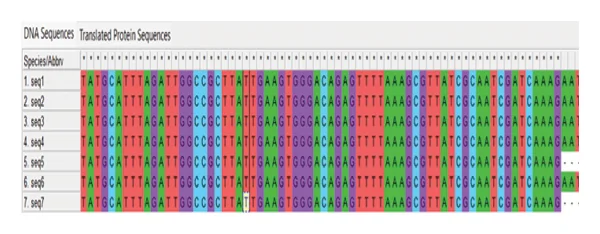
Multiple sequence alignment of seven *Staphylococcus aureus* isolates numbered 1 to 7. The aligned region is around 90 nucleotides long (positions 1–90 are displayed). The seven isolates share the same sequences at the aligned region, there were no changes of nucleotide sequences, insertions, and deletions found.

**FIGURE 4:**
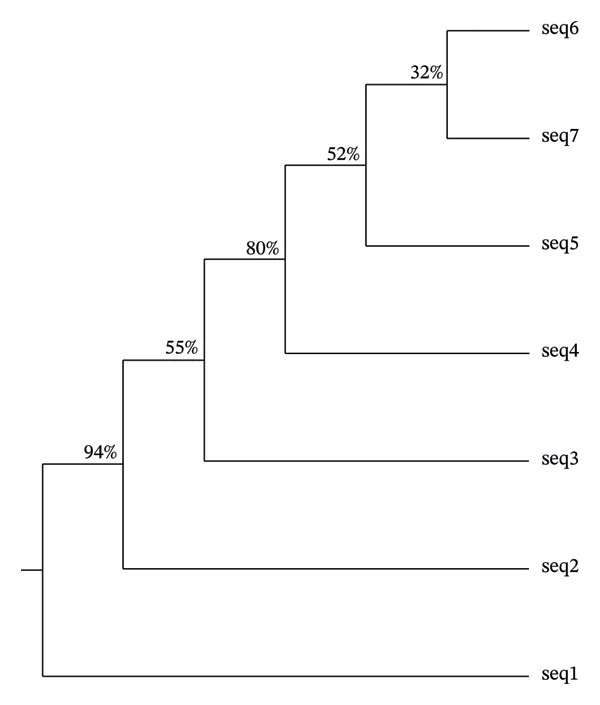
Phylogenetic tree of local isolates and percentage sequence identity matrix of seven *Staphylococcus aureus* isolates (seq1–7). The values are double nucleotide sequence identities over the aligned area between the sequences: 94% with seq2, 55% with seq3, 80% with seq4, 52% with seq5, 32% with seq6, and 32% with seq7. The genetic relatedness among the isolates varies greatly, as suggested by the identity values being 32%–94%. The highest identity is between seq1 and seq2 (94%), and the lowest is between seq1 and seq6 and seq1 and seq7 (32%).

**FIGURE 5:**
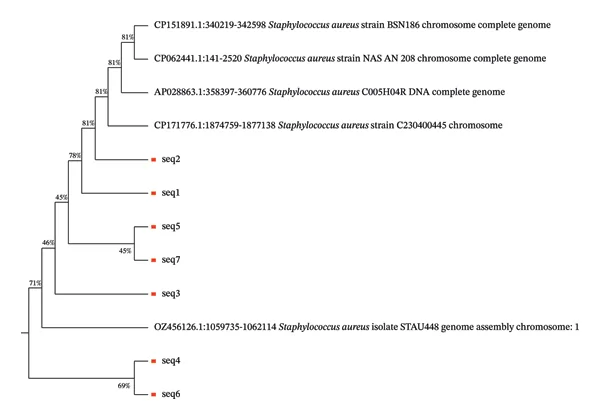
Phylogenetic comparison of local isolates of *S. aureus* (seq1–seq7) to international reference strains (81%–78% identity).

**FIGURE 6:**
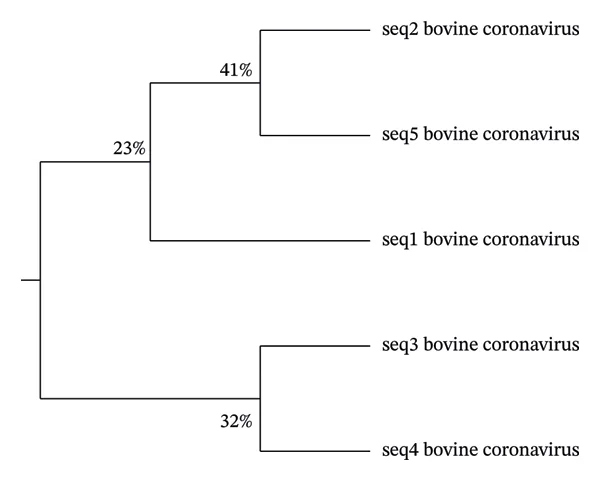
Local bovine coronavirus isolates (seq1–seq5) grouped into a phylogenetic tree using the *N* gene nucleotide sequences. The sequence identities between local isolates range from 23% to 41%, suggesting significant sequence divergence among isolates in the local population; seq2 is 41% identical to seq5, and 32% and 23% identical to seq1 and seq3, respectively. The low sequence identities imply high genetic diversity of circulating local bovine coronavirus strains.

**FIGURE 7:**
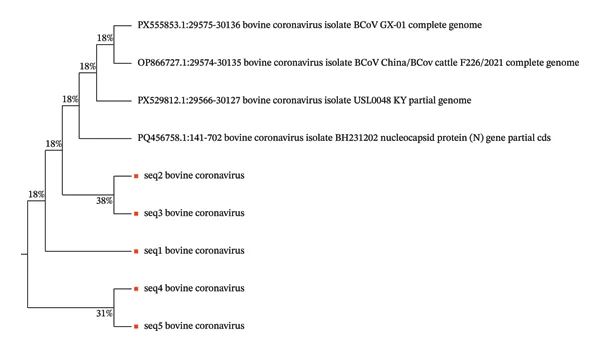
Sequences of local BCV isolates of the *N* gene aligned with international BCV isolates (seq1–seq5 sequences from this study, 18%–38% identity).

The colonies on the nutrient and selective media were golden yellow and typical of *S. aureus*, and Gram staining revealed Gram‐positive cocci arranged in grape‐like clusters (Figure 1). The BCoV *N* gene (205 bp) was confirmed by PCR (Figure 2), together with amplification of the 16S rRNA gene of *S. aureus* (1500 bp). Multiple sequence alignment of the local *S. aureus* isolates revealed highly conserved nucleotide regions (Figure 3). Phylogenetic analysis demonstrated considerable genetic variability among the local *S. aureus* isolates, while comparison with international reference strains showed their phylogenetic relationships and sequence divergence (Figures 4 and 5). Phylogenetic analysis of the local BCoV isolates revealed considerable genetic diversity (Figure 6), and sequence alignment with international reference strains further demonstrated nucleotide divergence among the local BCoV isolates (Figure 7).

The biochemical characteristics of the *S. aureus* isolates are summarized in Table 4.

**TABLE 4:** Biochemical data for bacterial isolates.

Code	Test	Result	Code	Test	Result	Code	Test	Result
AMY	Amygdalin	—	28	AlaA	—	54	MBdG	+
4	PIPLC	+	29	TyrA	—	56	PUL	—
5	dXYL	—	30	dSOR	—	57	dRAF	—
8	ADH1	+	31	URE	—	58	O129R	+
9	BGAL	—	32	POLYB	+	59	SAL	—
11	AGLU	+	37	dGAL	—	60	SAC	+
13	APPA	—	38	dRIB	+	62	dTRE	+
14	CDEX	—	39	ILATk	+	63	ADH2s	—
15	AspA	—	42	LAC	—	64	OPTO	+
16	BGAR	—	44	NAG	+			
17	AMAN	—	45	dMAL	+			
19	PHOS	(−)	46	BACI	+			
20	LeuA	+	47	NOVO	—			
23	ProA	—	50	NC6.5	(+)			
24	BGURr	—	52	dMAN	—			
25	AGAL	—	53	dMNE	—			
26	PyrA	+						
27	BGUR	—						

## 4. Discussion

In the present study, BCoV was isolated from 35% of the examined calves and *S. aureus* was isolated from 28% of the calves, with 15% of the calves being co‐infected with both pathogens (Table 1). These results suggest that both viral and bacterial infections play important roles in the epidemiology of BRD and enteric infections in young calves. In recent epidemiological studies of respiratory and gastrointestinal infections of calves, BCoV was also found to be widely prevalent in young cattle (less than 3 months of age) [17, 28]. The current result corroborates with Shin et al. [17], who reported the presence of BCoV in all calves with respiratory and diarrheic signs. Similarly, Chae et al. [28] suggested BCoV as a significant etiological cause of calf diarrhea and respiratory disease in Korean cattle.

The prevalence of *S. aureus* was fairly high in this study (Table 1), and recent data indicate that this pathogen is associated with respiratory infections, as well as with bovine mastitis. *S. aureus* can produce several virulence factors that are important for its ability to colonize and persist in respiratory tissues, such as hemolysins, adhesins, leukocidins, and biofilm formation [29, 30]. Wolf et al. [31] showed that *S. aureus* isolates obtained from the respiratory system of infected cattle tend to have enhanced virulence attributes and antimicrobial resistance, reinforcing its role in the pathogenesis of BRD.

The relatively high co‐infection rate observed in the present study suggests a possible interaction between BCoV and *S. aureus* infections (Table 1). Other studies have shown that viral‐bacterial synergism is a key factor in causing severe respiratory disease and recovery periods [7, 15]. Viral infection leads to injury of the respiratory epithelial cells, lowering innate immunity, and allowing bacteria to colonize [32]. Kim et al. (2021) have shown experimentally that calves co‐infected with BCoV and *S. aureus* had more severe bronchointerstitial pneumonia and greater inflammatory responses than those infected with just one of the pathogens. Hence, the high level of severity of the respiratory and enteric symptoms seen in the present study could be related to the combined effect of both viral and bacterial pathogens [33, 34].

A very significant association of BCoV positivity with the severity of the disease was found (Table 2). The prevalence rates of BCoV in acute and moderate clinical cases were 93.75% and 90%, respectively, with all mild clinical cases being negative (*p* < 0.001). Shin et al. [17] reported that there was a significantly higher percentage of calves with severe respiratory and enteric manifestations that tested positive for BCoV. Results suggest that BCoV infection may contribute to the development of more severe clinical manifestations.

Clinical signs noted in positive calves (fever, watery diarrhea, difficulty breathing, cough, and dehydration (Figure 1)) are in line with the pathogenesis of BCoV. Park et al. [7] showed that BCoV infects the epithelial cells of the respiratory and intestinal tract, leading to the destruction of the epithelium and inflammation and disrupting mucociliary clearance. These pathological changes provide an opportunity for secondary bacterial infection and exacerbate disease.

Neglecting BCoV from the mild cases also points to other pathogens, environmental factors, or partial maternal immunity to be associated with mild clinical disease [35]. Maternal antibodies passed on in colostrum have been proven to inhibit coronavirus replication and limit damage to tissues early in the calf’s development [36, 37].

However, this increased severity seen in co‐infected calves could also be due to interactions between viral and bacterial pathogens at the immunological level. Viral infections may impair innate immune responses, including alterations in macrophage and neutrophil functions, which can facilitate secondary bacterial colonization [38]. At the same time, *S. aureus* toxins cause necrosis and inflammation of tissue, leading to a vicious circle of tissue damage in the lungs and gut [39].

Bacteriological data confirmed *S. aureus* isolates. Colonies on nutrient agar and mannitol salt agar were circular, smooth, convex, opaque, and gold‐colored in appearance (Figure 2). The Gram stain revealed Gram‐positive cocci in grape‐like clusters, which are the typical characteristics of *S. aureus*. Classical/phenotypic microbiological methods were reliable with molecular confirmation, as reported by Gumaa et al. [12]. The biochemical profile as obtained in this study (Table 3) also confirmed the identification of the bacteria. Positive reactions of ADH1, AGLU, PyrA, POLYB, dMAL, and BACI were found to be consistent with the metabolic characteristics of *S. aureus*. Mannitol fermentation is commonly associated with the identification of pathogenic staphylococci and may reflect metabolic characteristics linked to virulence [40]. The level of variability found within biochemical reactions can suggest that there is a genetic diversity of circulating strains [41].

The N gene of BCoV was amplified to 205 bp using PCR, which resulted in the molecular detection of BCoV (Figure 3). Due to its diagnostic reliability and its importance in evolution, the N gene is one that has been widely used in molecular epidemiological studies [17]. One of the most sensitive methods available for the detection of coronavirus is real‐time RT‐PCR, which is able to detect the viral RNA even in the early stages of infection. Cho et al. [16] emphasized the need for multiplex molecular assays for fast and precise identification of the pathogens responsible for calf diarrhea.

Likewise, the amplification of the gene encoding the 16S rRNA gene of *S. aureus* at ∼1500 bp (Figure 4) demonstrated the reliability of molecular identification methods. The 16S rRNA gene is a very conserved gene among bacteria and is commonly used for bacterial taxonomy and phylogenetic analysis [42]. Sequencing of amplified products enables accurate identification and comparison with strains from different geographic regions [43].

The seven *S. aureus* isolates were compared by multiple sequence alignment of the regions of the nucleotides, and there were highly conserved regions with no insertions, deletions, or substitutions (Figure 5). Conserved sequence regions are frequently found in closely related variants that are all within the same strain population and/or environmental context [12]. A high percentage of similarity between local isolates may indicate the circulation of closely related strains within the study area [12]. However, phylogenetic analysis showed more genetic diversity among *S. aureus* isolates (Figure 6) despite the fact that there is sequence conservation in the region analyzed. Sequence similarity among the isolates ranged from 32% to 94%, indicating variable genetic relatedness. The highest similarity was found between seq1 and seq2, which implies that they are closely related; lower similarities suggest that other isolates diverged from each other. This diversity can be caused by mutation, horizontal gene transfer, adaptation in the environment, and antimicrobial pressure [44]. Recent research on *S. aureus* associated with cattle has found similar results for other geographic areas [45].

Based on the phylogenetic comparison of local isolates and international reference strains, there are nucleotide identities ranging from 78% to 81% (Figure 6). The results suggest that local isolates may possess region‐specific genetic characteristics. The observed divergence may reflect differences in management practices, antimicrobial use, or environmental conditions, although these factors were not investigated in the present study [46].

The local BCoV strains showed marked genetic variability among circulating BCoV strains, as revealed by the phylogenetic analysis (Figure 7). Sequences were often very different with identities between 23% and 41%. The highest similarity was found between seq2 and seq5, while the lowest similarity was found between seq2 and seq3. The RNA viruses are coronaviruses, which have high levels of mutation and recombination because they have inefficient proofreading during RNA replication [7, 17]. This genetic heterogeneity allows for quick adaptation to host immune pressure and to environmental changes.

The nucleotide identities between local BCoV isolates and international reference strains were 18%–38% (Figure 7). These findings indicate that there are local viral lineages that circulate. In recent studies from Asia and Europe, there has been similar genetic diversity, with several BCoV strains that were detected within small geographic areas [47, 48].

The genetic diversity observed has significant implications in diagnosis and vaccines. Viral mutations could change the antigenicity and therefore the efficacy of the vaccine, and mutations in the primer‐binding regions may affect the sensitivity of the PCR [17]. It is therefore crucial to conduct molecular surveillance continuously to update diagnostic assays, track viral variants, and ensure effective and efficient monitoring.

The present findings support a possible synergistic interaction between BCoV and *S. aureus* in the pathogenesis of BRD. Co‐infected calves exhibited more severe clinical signs than those infected with a single pathogen (Table 2). Viral‐induced epithelial damage and impaired mucociliary clearance may facilitate bacterial adhesion and colonization, whereas *S. aureus* virulence factors, including adhesins, hemolysins, leukocidins, and enterotoxins, may further amplify tissue injury and inflammation, resulting in more severe respiratory and enteric lesions [32, 49–51].

In addition to its relatively high prevalence, *S. aureus* is increasingly recognized as an important respiratory pathogen in cattle, particularly in the presence of concurrent viral infections [9, 13]. Its ability to colonize, evade host immunity, and damage tissues is attributed to several virulence factors, including adhesins, hemolysins, leukocidins, and biofilm‐associated proteins [10]. These findings provide epidemiological evidence for the circulation of genetically diverse BCoV and *S. aureus* strains among calves with BRD. Despite its evolution over the years and the development of new treatments, BRD continues to be one of the most critical economic issues in cattle production worldwide, leading to high production losses via treatment costs, reduced growth, and cow deaths [7, 28]. Young calves are especially vulnerable due to their immature immune systems and environmental factors like overcrowding, transportation, poor air quality, and not consuming sufficient colostrum [52].

The detection of genetically variable BCoV and *S. aureus* strains suggests the presence of diverse circulating strains within the study population, which might make disease control more difficult. Another potential concern with the presence of *S. aureus* is the issue of antimicrobial resistance, as the amount of horizontal gene transfer of antimicrobial resistance genes is high in livestock‐associated strains [53].

A One Health perspective may be valuable for understanding the ecology of respiratory pathogens in livestock populations. Although BCoV primarily infects cattle, continuous molecular surveillance is important because viral mutation and recombination may influence pathogen evolution and epidemiological patterns [54]. In addition, monitoring *S. aureus* in livestock remains important due to its clinical significance in animal health and the need to understand the evolution of antimicrobial resistance and pathogen characteristics [55].

## 5. Conclusion

This study demonstrated that BCoV and *S. aureus* are important pathogens associated with BRD in Iraqi calves, with co‐infection being significantly associated with increased clinical severity. Molecular characterization and phylogenetic analysis revealed considerable genetic diversity among local BCoV and *S. aureus* isolates, indicating the circulation of genetically heterogeneous strains within the study population. The association between co‐infection and severe clinical manifestations, together with the observed molecular variability of both pathogens, provides valuable epidemiological and molecular evidence for understanding the pathogenesis and circulation of BRD‐associated pathogens under field conditions in Iraq. These findings highlight the importance of integrating clinical assessment with molecular diagnostics and phylogenetic surveillance to improve pathogen detection, monitor the emergence and evolution of circulating strains, and support more effective BRD surveillance and control programs. Further multicenter studies involving larger sample sizes are warranted to better define the epidemiology, genetic evolution, and clinical significance of BCoV–*S. aureus* co‐infection in cattle.

## Author Contributions

Noor R. Abady: research concept and study design, manuscript writing, critical revision, and final approval of the article. Karrar Jasim Hamzah: data collection and laboratory work. Sumod Abdul Kadhem Salman: data analysis and statistical evaluation. Diyar Khlaif Flaifel: interpretation of results. Dhay Ali Abed: conducted data validation, and Ahmed Hamzah Mosa: performed clinical signs examination.

## Funding

This work was self‐funded by the authors and has not received any external funding. The source of financial support did not influence the study design, data collection, data analysis, data interpretation, writing of the manuscript, or the decision to submit it for publication.

## Disclosure

All authors read and approved the final manuscript. The corresponding author had access to all the data in this study and is accountable for the integrity of the data and the accuracy of the data analysis.

The relevant author confirms that this manuscript is an accurate, complete, and honest report of the study reported; that no important aspect of the study has been omitted; and that any departures from the study as planned have been explained.

## Conflicts of Interest

The authors declare no conflicts of interest.

## Data Availability

All data used in this study are available in the manuscript and are available upon reasonable request from the authors.
